# NARRATIVES ENHANCING TRAINEES’ ATTITUDES TOWARD AGING

**DOI:** 10.1093/geroni/igz038.2103

**Published:** 2019-11-08

**Authors:** James S Powers

**Affiliations:** VANDERBILT UNIVERSITY SCHOOL OF MEDICINE, Nashville, Tennessee, United States

## Abstract

We analyzed narratives from graduate students trained in aging themes who then interviewed older hospitalized adults about their life experiences. Three Discovery Fellows given a 90-minute training on open-ended interviewing, the medical care system, empathy, and aging themes derived from the Reframing Aging Program performed semi-structured interviews of 30-90 minutes. Subjects were hospitalized older veterans receiving care by an interdisciplinary geriatric rehabilitation team. Narratives were composed following the interviews and electronically uploaded to a secure site. Qualitative analysis was performed with each statement treated as a separate quote using a hierarchical coding system. A total of 774 codes were used to describe the themes present in 582 quotes. A conceptual framework from the narrative themes emerged with 5 groupings: 1) personal experience, 2) people important in life, 3) self-reflection, 4) medical condition, and 5) health maintenance. Concepts displayed included openness, compassion, and morality, as components of wisdom.

